# Association between Semen Exposure and Incident Bacterial Vaginosis

**DOI:** 10.1155/2011/842652

**Published:** 2011-12-08

**Authors:** Maria F. Gallo, Lee Warner, Caroline C. King, Jack D. Sobel, Robert S. Klein, Susan Cu-Uvin, Anne M. Rompalo, Denise J. Jamieson

**Affiliations:** ^1^Division of Reproductive Health, Centers for Disease Control and Prevention, 4770 Buford Highway, Mail Stop K-34, Atlanta, GA 30341-3724, USA; ^2^Department of Medicine, Wayne State University School of Medicine, Harper Hospital, 2990 John R. Street, 5 Hudson, Detroit, MI 48201, USA; ^3^Mount Sinai School of Medicine, One Gustave L. Levy Place, Box 1057, New York, NY 10029, USA; ^4^Brown University School of Medicine, The Miriam Hospital, 164 Summit Avenue, Providence, RI 02906, USA; ^5^Johns Hopkins Bayview Medical Center, Johns Hopkins University School of Medicine, 5200 Eastern Avenue, MFL Building, Center Tower, Suite 4000, Baltimore, MD 21224, USA

## Abstract

*Objective*. To identify correlates of incident bacterial vaginosis (BV) diagnosed with Nugent scoring among high-risk women. *Study Design*. We conducted both cohort and case-crossover analyses, stratified by HIV infection status, based on 871 HIV-infected and 439 HIV-uninfected participants in the HIV Epidemiology Research Study, conducted in 4 US sites in 1993–2000. *Results*. BV incidence was 21% and 19% among HIV-infected and -uninfected women, respectively. Fewer correlates of BV were found with case-crossover than with cohort design. Reporting frequent coitus (regardless of consistency of condom use) was correlated with BV in cohort analyses but not in case-crossover analyses. The sole correlate of BV in both types of analyses was the detection of spermatozoa on Gram stain, which is a marker of semen exposure. *Conclusion*. The inconsistent association between condom use and BV in prior studies could be from reporting bias. We found evidence of a relationship between semen exposure and incident BV.

## 1. Introduction

Bacterial vaginosis (BV) is a common vaginal condition with an estimated prevalence of 29% among U.S. women during 2001–2004 [1]. BV has been linked to a range of adverse reproductive outcomes, including infertility, spontaneous abortion, preterm premature rupture of the membranes, amniotic fluid infection, low birth weight, and preterm delivery [2–8]. BV also might increase women's risk for pelvic inflammatory disease although evidence on this possible association is inconsistent [9–11]. In addition, evidence suggests that BV increases women's risk for sexually transmitted infections (STIs), including gonorrhea, chlamydial infection, trichomoniasis, human papillomavirus, herpes simplex virus, and HIV [12–19]. 

Although the etiology of BV remains unknown, two competing hypotheses currently prevail [20–22]. In the first, BV is viewed as an imbalance of the vaginal microbiota caused by the colonization of endogenous organisms from the intestinal tract [23]. This imbalance could be precipitated by a variety of events, including coitus and vaginal cleansing or douching. The second hypothesis holds that BV is caused by the sexual transmission of a specific pathogen (e.g., *Gardnerella vaginalis *or unknown bacteria). The similarity between the epidemiology of BV and that of STIs supports the hypothesis that BV is sexually transmitted. For example, BV has been associated with risky sexual behaviors, including having new or a relatively high number of sexual partners, having sex frequently, not using condoms, using drugs during sex, and having sex with uncircumcised partners [1, 16, 22, 24, 25]. However, because these associations often have been found in observational studies, they could be the result of uncontrolled confounding. Use of a case-crossover analysis (in which each woman serves as her own control) would minimize the effects of time-independent confounders [25]. We conducted both cohort and case-crossover analyses to identify time-variant correlates of BV among a cohort of high-risk women in the U.S., who participated in a longitudinal study of the effects of HIV infection on women's health [26].

## 2. Materials and Methods

We analyzed data from the HIV Epidemiology Research Study (HERS), which was conducted at 4 U.S. sites (Bronx, NY; Detroit, MI; Baltimore, MD; and Providence, RI, USA) in 1993–2000 [26]. Participants consisted of 871 HIV-infected women and 439 uninfected women who, at the time they enrolled in the study, were 16–55 years of age, did not have an AIDS-defining clinical diagnosis, and either injected drugs or engaged in high-risk sexual behaviors (i.e., had >5 sexual partners in the previous 5 years, traded sex for money or drugs, or had sex with a male who injected drugs or who was suspected of being or known to be infected with HIV). After enrollment, participants completed follow-up visits scheduled at 6-month intervals. During these visits, HERS staff conducted interviews to collect demographic, health, and behavioral information, conducted physical examinations, and collected specimens to be tested for infections, including BV, HIV, human papillomavirus (HPV), and trichomoniasis. Study visits were not used to diagnose or treat symptoms, and less than 1% of participants reported using metronidazole or topical clindamycin [27]. Ethical review boards at the study sites and the Centers for Disease Control and Prevention approved the study, and only women who gave informed consent were enrolled.

Gram-stained slides prepared from swabs of posterior vaginal fornix specimens were air dried, fixed in methanol, and shipped to a central laboratory where a single technician used oil immersion with ×1000 magnification to quantify and score the specimens. Specimens with a Nugent score of 7–10 were considered positive for BV [28]. Gram stains also were evaluated for morphological identification of spermatozoa, which is specific for recent exposure to semen [29]. Spermatozoa usually clear from vaginal secretions by 12–36 hours after exposure to semen although they have been detected microscopically up to 10 days after exposure [30, 31]. Wet mount was used for diagnosing trichomoniasis, and vaginal specimens were cultured for *Candida *organisms. Aliquots of cervicovaginal lavage fluid were frozen for later testing for HPV by polymerase chain reaction.

We limited our analyses to data collected during participants' first 10 follow-up visits and excluded the 12 women who HIV seroconverted during the study. Participants' incident BV status was assessed at follow-up visits only if Nugent scores of samples collected at their preceding visit indicated that they were BV negative. If they tested positive for BV or their Nugent scores were missing, their incident BV status was coded as missing. We used unconditional (using generalized estimating equations to account for intrasubject correlation from multiple visits) and conditional logistic regression to analyze the data as if they were derived from a cohort and case-crossover study, respectively. For both analyses, we constructed individual models to evaluate the correlates of incident BV for HIV-infected and -uninfected women separately. While the analytic population for the cohort analysis included all follow-up visits with nonmissing data on incident BV, the case-crossover analysis was limited to follow-up visits from women who had ≥1 follow-up visit *with *and ≥1 follow-up visit *without *incident BV. 

For both the cohort and case-crossover analyses, we fitted individual models to assess the bivariable relationship between each potential correlate and incident BV. For the multivariable analyses, we fitted full models with all potential correlates and used manual, backward elimination to exclude factors that were not significantly associated (based on an alpha of 0.05) with incident BV. Potential correlates were selected because of their prior identification in the literature. The cohort analyses included both time-independent and -dependent variables. However, because individual participants in the case-crossover analyses served both as case subjects and matching control subjects, the variables evaluated in these analyses were limited to time-dependent factors, which had the potential to vary between the participant's visits.

## 3. Results

Because of the differences in findings by HIV status in both the cohort and case-crossover analyses, we present results separately for HIV-infected and -uninfected participants. The cohort analyses were based on data collected during 3,050 visits by 799 HIV-infected women and 1,564 visits by 375 uninfected women. The case-crossover analyses were based on data collected during 1,543 visits by 332 HIV-infected women and 753 visits by 159 uninfected women. The incidence of BV during the study follow-up period was 21% among HIV-infected women and 19% among uninfected women. 

### 3.1. HIV-Infected Participants

The four time-independent variables assessed (i.e., study site, age at baseline, race, and education at baseline) were significantly associated with incident BV in the bivariable, cohort analyses among HIV-infected women (Table 1). All variables except for study site were also significantly associated with incident BV in the multivariable analysis, the results of which showed risk for incident BV to be higher among women younger than 45 years of age than among those older, higher among black women than among white women, and higher among women with a high-school education or less than among those with post-high-school education. Seven time-dependent variables were correlated with incident BV in the bivariable, cohort analyses. Except for current injection drug use and cigarette use within six months, these also were associated with incident BV in the multivariable, cohort analysis. Visits with trichomoniasis at the preceding visit (adjusted odds ratio [aOR], 1.8; 95% confidence interval [CI], 1.4–2.4), HPV at the preceding visit (aOR, 1.3; 95% CI, 1.0–1.5), and spermatozoa detected (aOR, 1.5; 95% CI, 1.1–2.1) had more incident BV than visits without these diagnoses. Also, visits in which the woman reported inconsistent condom use and frequent coitus (aOR, 1.6; 95% CI, 1.2–2.2) or consistent condom use and frequent coitus (aOR, 1.6; 95% CI, 1.2–2.1) were associated with more incident BV than visits in which women reported no sexual activity. Finally, crack use within six months correlated with incident BV (aOR, 1.9; 95% CI, 1.4–2.6). 

The bivariable, case-crossover analyses yielded four correlates of incident BV among HIV-infected women: spermatozoa detection, sexual behaviors, current injection drug use, and crack use within six months (Table 2). Except for sexual behaviors, these variables remained significantly associated in the multivariable, case-crossover analysis. Visits with spermatozoa detection (aOR, 1.6; 95% CI, 1.1–2.5), reports of current injection drug use (aOR, 1.9; 95% CI, 1.1–3.3) and reports of crack use within six months (aOR, 1.6; 95% CI, 1.0–2.7) were more likely to have incident BV than visits without these factors.

### 3.2. HIV-Uninfected Participants

Among HIV-uninfected participants, study site and race were the only time-independent variables significantly associated with BV risk in the bivariable, cohort analyses, and both remained significantly associated with BV risk in the multivariable, cohort analysis (Table 3). Black women had a higher risk than women of other races (aOR, 1.9; 95% CI, 1.3–2.7). Results of the bivariable, cohort analyses results showed six time-dependent variables to be significantly associated with incident BV risk. All except one (crack use within the previous 6 months) also were associated with BV risk in the multivariable analyses. Factors significantly associated with incident BV risk in the multivariable analyses were trichomoniasis at the preceding visit (aOR, 1.7; 95% CI, 1.1–2.6), spermatozoa detection (aOR, 1.9; 95% CI, 1.3–2.9), coitus ≥4 times per month during the previous 6 months and either inconsistent condom use (aOR, 1.9; 95% CI, 1.3–2.8) or consistent condom use (aOR, 1.9; 95% CI, 1.2–3.1) cigarette use during previous 6 months (aOR, 1.5; 95% CI, 1.0–2.1), and current use of hormonal contraception (aOR, 0.4; 95% CI, 0.2–0.8). 

Two factors were associated with incident BV in the bivariable, case-crossover analyses among HIV-uninfected participants, and both remained associated in the multivariable analysis (Table 4). Visits with spermatozoa detected were more likely to have incident BV (aOR, 2.1; 95% CI, 1.1–4.0) than visits without its detection. Visits with self-reported frequent coitus and either inconsistent condom use (aOR, 3.0; 95% CI, 1.5–5.9) or consistent condom use (aOR, 3.1; 95% CI, 1.3–7.4) had more incident BV than visits with self-reported lack of sexual activity.

## 4. Discussion

The sole correlate of incident BV that emerged in both the cohort and case-crossover analyses among HIV-infected and -uninfected women was the detection of spermatozoa on Gram stain, which is a biological marker of recent exposure to semen. The cohort analyses among HIV-infected and -uninfected women also found that incident BV was more common among those reporting frequent coitus (regardless of the consistency of condom use); however, this association was not found in the case-crossover analyses. A protective effect of condoms against BV has been demonstrated in some prior studies (including a case-crossover analysis), but not in all studies [22, 32–34]. The failure to find a relationship between condom use and decreased BV risk in earlier studies could have been the result of misclassification in participant reporting of coitus and condom use. This misclassification could have occurred if studies collected inaccurate reports of condom use, as a result of social desirability or recall bias, or did not collect comprehensive data on condom use, including possible malfunctions or misuse. A protective effect of condoms against BV also could have been obscured in previous studies because of the role of recurring cases of BV. That is, if unprotected coitus can cause incident BV but is not a necessary component for its recurrence, establishing the link between unprotected coitus, and incident BV could be difficult. 

We found fewer correlates of incident BV in our case-crossover analyses than in our cohort analyses. Results of the adjusted case-crossover analyses of incident BV among HIV-infected women showed only spermatozoa detection, current injection drug use, and crack use within the previous 6 months to be associated with incident BV, whereas results of the cohort analyses among HIV-infected women also showed trichomoniasis at the previous visit, HPV at the previous visit and coitus ≥4 times per month during the previous 6 months to be associated with incident BV. Similarly, results of the case-crossover analyses of risk among HIV-uninfected women only found spermatozoa detection and coitus ≥4 times per month during the previous 6 months to be associated with incident BV, whereas results of the cohort analyses among HIV-uninfected women also showed trichomoniasis at the previous visit, current hormonal contraception use, and cigarette use within the previous 6 months to be associated with incident BV. The case-crossover analyses might have identified fewer correlates of incident BV as a result of reduced confounding from each woman serving as both a case subject (visits with incident BV) and a matching control subject (visits without incident BV) [25]. Alternatively, reduced power in the case-crossover analyses might have prevented the detection of correlates of BV.

A major study limitation was that BV was only assessed at six-month intervals. Studies with frequent sampling have suggested that women may have rapid fluctuations in vaginal microbiota, including short episodes of BV that resolve spontaneously [35, 36]. Thus, our study might have missed cases of BV. We were also unable to determine the temporal relationship between exposure to semen and the development of BV; as a result, we cannot rule out the possibility that the association between the two factors is the result of BV causing longer persistence of spermatozoa in vaginal fluid rather than semen exposure actually causing BV. Previous case-crossover analyses also suggest that recent menses, use of vaginal lubricants, rectal sex, douching for cleansing after menstruation, and psychosocial stress could be risk factors for incident BV [36–38]. None of these factors, though, were evaluated in the present analysis. Finally, although the detection of spermatozoa is specific for recent exposure to semen, it is not a sensitive marker and cases of exposure might have been missed [29, 39]. Strengths of our study included our use of data from a large, prospective study in which semen exposure was assessed by an objective measure and our use of case-crossover analyses, which allowed us to reduce possible effects of unmeasured time-independent confounding by comparing women to their own control visits. 

The detection of BV among women who have reported being sexually abstinent has been an argument against the role of sexual activity as a necessary component in causing BV [40, 41]. However, results of a recent study among young adults with a laboratory-diagnosed case of chlamydial infection, gonorrhea, or trichomoniasis showed that 10% reported having abstained from penile-vaginal intercourse in the previous year and that 6% reported never having had intercourse [42]. Thus, imperfect validity of self reports could explain the occurrence of BV among women reporting abstinence in prior studies. While study findings implicate the role of sexual exposure in the development of incident BV, this does not necessarily mean that BV is caused by the transmission of specific organism(s) during intercourse. Semen exposure could also increase women's risk for incident BV by increasing vaginal pH levels, changing the growth patterns in bacteria populations, or exposing women to an unidentified component of semen. The present study found biological evidence of an association between semen exposure and incident BV, which provides new support for the sexual transmission of BV; however, the mechanism remains unknown. 

## Figures and Tables

**Table 1:** Results of cohort analyses of associations between selected factors and incident bacterial vaginosis among HIV-infected U.S. women, HERS, 1993–2000*.

	Control visits	Case visits	Bivariable model		Multivariable model^†^	
	No.	No.	OR	(95% CI)	aOR	(95% CI)
*Time-independent factors*		
Study site		
Site 1	749	138	Referent		Referent	
Site 2	502	170	1.8	(1.3, 2.4)	1.3	(0.9, 1.8)
Site 3	410	173	2.4	(1.7, 3.2)	1.4	(1.0, 2.0)
Site 4	761	147	1.1	(0.8, 1.5)	1.1	(0.8, 1.6)
Age at baseline (in years)						
16–24	43	16	2.2	(1.1, 4.3)	1.4	(0.7, 3.0)
25–34	664	217	2.2	(1.6, 3.0)	2.0	(1.4, 2.8)
35–44	1235	324	1.7	(1.3, 2.3)	1.6	(1.2, 2.1)
45+	480	71	Referent		Referent	
Race						
Black	1239	430	2.1	(1.6, 2.6)	1.7	(1.3, 2.3)
Other	1183	198	Referent		Referent	
Education at baseline						
<High school	906	289	1.8	(1.4, 2.4)	1.6	(1.2, 2.1)
High school	763	209	1.5	(1.2, 2.0)	1.5	(1.1, 1.9)
>High school	750	129	Referent		Referent	

*Time-dependent factors*						
Visit (0–10)			0.9	(0.9, 1.0)	1.0	(0.9, 1.0)
CD4+ group (cells/*μ*L)						
0–199	567	147	1.0	(0.8, 1.3)		
200–499	1213	308	1.1	(0.9, 1.5)		
≥500	598	166	Referent			
Trichomoniasis at preceding visit						
Yes	180	117	2.2	(1.7, 2.8)	1.8	(1.4, 2.4)
No	2240	510	Referent		Referent	
Vaginal *Candidal* culture at preceding visit						
Yes	804	247	1.2	(1.0, 1.4)		
No	1612	377	Referent	
Human papillomavirus at preceding visit						
Yes	1468	432	1.3	(1.0, 1.5)	1.3	(1.0, 1.5)
No	934	188	Referent		Referent	
Spermatozoa detected on Gram stain						
Yes	139	70	1.7	(1.3, 2.4)	1.5	(1.1, 2.1)
No	2283	558	Referent		Referent	
Sexual behavior during previous 6 months^‡^						
Frequent coitus, inconsistent condom use	331	127	2.0	(1.6, 2.7)	1.6	(1.2, 2.2)
Frequent coitus, consistent condom use	431	146	1.8	(1.4, 2.4)	1.6	(1.2, 2.1)
Infrequent coitus, inconsistent condom use	240	65	1.4	(1.0, 1.9)	1.2	(0.9, 1.7)
Infrequent coitus, consistent condom use	488	127	1.4	(1.1, 1.8)	1.3	(1.0, 1.7)
Not sexually active	920	162	Referent		Referent	
Female sex partner during previous 6 months						
Yes	86	25	1.1	(0.8, 1.7)		
No	2320	601	Referent			
Douching within previous 48 hours						
Yes	72	24	1.0	(0.6, 1.6)		
No	2340	602	Referent			
Current hormonal contraception use						
Yes	101	29	1.2	(0.8, 1.8)		
No	2319	599	Referent			
Current injection drug use						
Yes	304	131	1.7	(1.4, 2.2)		
No	2114	497	Referent			
Crack use during previous 6 months						
Yes	199	117	2.2	(1.6, 2.9)	1.9	(1.4, 2.6)
No	2219	511	Referent		Referent	
Cigarette use during previous 6 months						
Yes	1639	484	1.5	(1.2, 1.9)		
No	779	144	Referent			

HERS = HIV Epidemiology Research Study; OR: odds ratio; aOR: adjusted odds ratio; CI: confidence interval.

*Findings from unconditional logistic regression model, using generalized estimating equations, based on 628 case visits (i.e., visits with incident bacterial vaginosis) and 2422 control visits (i.e., visits without incident bacterial vaginosis) completed by 799 participants.

^†^Adjusted for all variables in column.

^‡^Frequent coitus defined as ≥4 times per month and infrequent coitus defined as <4 times per month.

**Table 2:** Results of case-crossover analyses of associations between selected factors and incident bacterial vaginosis among HIV-infected U.S. women, HERS, 1993–2000*.

	Control visits	Case visits	Bivariable model		Multivariable model^†^	
	No.	No.	OR	(95% CI)	aOR	(95% CI)
*Time-dependent factors*						
CD4+ group (cells/*μ*L)						
0–199	257	112	0.8	(0.4, 1.3)		
200–499	544	228	0.8	(0.5, 1.2)		
≥500	249	128	Referent			
Trichomoniasis at preceding visit						
Positive	100	74	1.2	(0.8, 1.9)		
Negative	967	400	Referent	
Vaginal *Candidal* culture at preceding visit						
Positive	371	181	1.1	(0.9, 1.5)		
Negative	695	290	Referent	
Human papillomavirus at preceding visit						
Positive	668	326	0.8	(0.6, 1.2)		
Negative	392	143	Referent			
Spermatozoa detected on Gram stain						
Yes	70	48	1.5	(1.0, 2.3)	1.6	(1.1, 2.5)
No	998	427	Referent		Referent	
Sexual behavior during previous 6 months^‡^						
Frequent coitus, inconsistent condom use	151	92	1.9	(1.2, 3.1)		
Frequent coitus, consistent condom use	177	113	1.7	(1.1, 2.8)		
Infrequent coitus, inconsistent condom use	118	46	1.2	(0.7, 2.0)		
Infrequent coitus, consistent condom use	225	96	1.2	(0.8, 1.8)		
Not sexually active	388	127	Referent	
Female sex partner during previous 6 months						
Yes	51	20	1.5	(0.6, 3.9)		
No	1010	453	Referent			
Douching within 48 hours						
Yes	38	19	0.7	(0.4, 1.3)		
No	1025	454	Referent			
Current hormonal contraception use						
Yes	30	23	2.4	(0.9, 6.7)		
No	1037	452	Referent			
Current injection drug use						
Yes	159	97	2.1	(1.2, 3.5)	1.9	(1.1, 3.3)
No	907	378	Referent		Referent	
Crack use during previous 6 months						
Yes	107	81	1.8	(1.1, 2.9)	1.6	(1.0, 2.7)
No	959	394	Referent		Referent	
Cigarette use during previous 6 months						
Yes	758	364	1.4	(0.7, 2.7)		
No	308	111	Referent			

*Findings from conditional logistic regression model based on 475 case visits (i.e., visits with incident bacterial vaginosis) and 1068 control visits (i.e., visits without incident bacterial vaginosis) completed by 332 women with at least one case and one control visit.

^†^Adjusted for all variables in column.

^‡^Frequent coitus defined as ≥4 times per month and infrequent coitus defined as <4 times per month.

**Table 3:** Results of cohort analyses of associations between selected factors and incident bacterial vaginosis among HIV-uninfected U.S. women, HERS, 1993–2000*.

	Control visits	Case visits	Bivariable model		Multivariable model^†^	
	No.	No.	OR	(95% CI)	aOR	(95% CI)
*Time-independent factors*						
Study site						
Site 1	437	65	Referent		Referent	
Site 2	185	77	2.7	(1.7, 4.2)	1.9	(1.2, 3.1)
Site 3	271	94	2.4	(1.5, 3.7)	1.7	(1.0, 2.7)
Site 4	372	63	1.1	(0.7, 1.7)	1.5	(1.0, 2.3)
Age at baseline (in years)						
16–24	51	7	0.9	(0.3, 2.1)		
25–34	386	106	1.4	(0.9, 2.2)		
35–44	597	139	1.1	(0.7, 1.6)		
45+	231	47	Referent			
Race						
Black	568	201	2.5	(1.8, 3.5)	1.9	(1.3, 2.7)
Other	697	98	Referent		Referent	
Education at baseline						
<High school	367	99	1.4	(1.0, 2.1)		
High school	436	101	1.1	(0.8, 1.6)		
>High school	457	99	Referent			

*Time-dependent factors*						
Visit (0–15)			0.9	(0.9, 1.0)	0.94	(0.9, 1.0)
Trichomoniasis at preceding visit						
Yes	100	60	2.1	(1.4, 3.2)	1.7	(1.1, 2.6)
No	1163	239	Referent		Referent	
Vaginal *Candidal* culture at preceding visit						
Yes	393	91	1.0	(0.8, 1.3)		
No	864	208	Referent	
Human papillomavirus at preceding visit						
Yes	278	84	1.3	(0.9, 1.7)		
No	975	210	Referent	
Spermatozoa detected on Gram stain						
Yes	76	43	2.3	(1.6, 3.3)	1.9	(1.3, 2.9)
No	1189	256	Referent		Referent	
Sexual behavior during previous 6 months^‡^						
Frequent coitus, inconsistent condom use	411	139	2.3	(1.6, 3.3)	1.9	(1.3, 2.8)
Frequent coitus, consistent condom use	129	35	1.8	(1.1, 2.9)	1.9	(1.2, 3.1)
Infrequent coitus, inconsistent condom use	244	49	1.4	(0.9, 2.0)	1.2	(0.8, 1.8)
Infrequent coitus, consistent condom use	138	23	1.1	(0.6, 1.9)	1.1	(0.6, 2.0)
Not sexually active	341	52	Referent		Referent	
Female sex partner during previous 6 months						
Yes	92	25	1.2	(0.7, 1.8)		
No	1169	272	Referent			
Douching within 48 hours						
Yes	44	13	1.1	(0.6, 2.0)		
No	1215	285	Referent			
Current hormonal contraception use						
Yes	92	9	0.4	(0.2, 0.9)	0.4	(0.2, 0.8)
No	1171	289	Referent		Referent	
Current injection drug use						
Yes	160	58	1.5	(1.0, 2.2)		
No	1104	241	Referent			
Crack use during previous 6 months						
Yes	139	66	2.0	(1.3, 2.9)		
No	1125	233	Referent			
Cigarette use during previous 6 months						
Yes	837	230	1.7	(1.2, 2.4)	1.5	(1.0, 2.1)
No	427	69	Referent		Referent	

*Findings from unconditional logistic regression model, using generalized estimating equations, based on 299 case visits (i.e., visits with incident bacterial vaginosis) and 1265 control visits (i.e., visits without incident bacterial vaginosis) completed by 375 participants.

^†^Adjusted for all variables in column.

^‡^Frequent coitus defined as ≥4 times per month and infrequent coitus defined as <4 times per month.

**Table 4:** Results of case-crossover analyses of associations between selected factors and incident bacterial vaginosis among HIV-uninfected U.S. women, HERS, 1993–2000*.

	Control visits	Case visits	Bivariable model		Multivariable model^†^	
	No.	No.	OR	(95% CI)	aOR	(95% CI)
Time-dependent factors						
Trichomoniasis at preceding visit						
Yes	76	41	1.2	(0.7, 2.1)		
No	446	189	Referent			
Vaginal *Candidal* culture at preceding visit						
Yes	143	70	1.1	(0.7, 1.6)		
No	377	160	Referent	
Human papillomavirus at preceding visit						
Yes	126	55	1.0	(0.6, 1.5)		
No	393	171	Referent			
Spermatozoa detected on Gram stain						
Yes	36	29	2.4	(1.3, 4.5)	2.1	(1.1, 4.0)
No	487	201	Referent		Referent	
Sexual behavior during previous 6 months‡						
Frequent coitus, inconsistent condom use	159	100	3.3	(1.7, 6.6)	3.0	(1.5, 5.9)
Frequent coitus, consistent condom use	46	29	3.1	(1.3, 7.3)	3.1	(1.3, 7.4)
Infrequent coitus, inconsistent condom use	114	36	1.7	(0.8, 3.4)	1.6	(0.8, 3.2)
Infrequent coitus, consistent condom use	58	19	1.1	(0.5, 2.4)	1.1	(0.5, 2.5)
Not sexually active	145	45	Referent		Referent	
Female sex partner during previous 6 months						
Yes	48	25	1.7	(0.6, 4.6)		
No	472	204	Referent			
Douching within 48 hours						
Yes	23	10	0.8	(0.3, 2.2)		
No	497	220	Referent			
Current hormonal contraception use						
Yes	22	8	0.4	(0.1, 1.3)		
No	500	221	Referent			
Current injection drug use						
Yes	93	42	1.5	(0.7, 3.2)		
No	429	188	Referent			
Crack use during previous 6 months						
Yes	93	45	1.4	(0.8, 2.5)		
No	429	185	Referent			
Cigarette use during previous 6 months						
Yes	373	173	1.4	(0.6, 3.5)		
No	149	57	Referent			

*Findings from conditional logistic regression model based on 230 case visits (i.e., visits with incident bacterial vaginosis) and 523 control visits (i.e., visits without incident bacterial vaginosis) completed by 159 women with at least one case and one control visit.

^†^Adjusted for all variables in column.

^‡^Frequent coitus defined as ≥4 times per month and infrequent coitus defined as <4 times per month.
